# Prescriptions for Codeine or Hydrocodone Cough and Cold Medications to US Children and Adolescents Following US Food and Drug Administration Safety Communications

**DOI:** 10.1001/jamanetworkopen.2021.34142

**Published:** 2021-11-11

**Authors:** Kao-Ping Chua, Rena M. Conti

**Affiliations:** 1 Department of Pediatrics, Susan B. Meister Child Health Evaluation and Research Center, University of Michigan Medical School, Ann Arbor; 2 Department of Health Management and Policy, University of Michigan School of Public Health, Ann Arbor; 3 Department of Markets, Public Policy, and Law, Institute for Health System Innovation and Policy, Boston University Questrom School of Business, Boston, Massachusetts

## Abstract

This cross-sectional study uses national prescription data to assess whether safety communications from the US Food and Drug Administration (FDA) issued in 2017 and 2018 were associated with changes in prescriptions for codeine and hydrocodone cough and cold medications to children and adolescents.

## Introduction

Cough and cold medications containing codeine or hydrocodone are associated with misuse and addiction in adolescents and with potentially fatal respiratory depression in young children.^1,2,3,4^ On April 20, 2017, the US Food and Drug Administration (FDA) issued a safety communication announcing a contraindication against all codeine use in children aged 0 to 11 years.^1^ On January 11, 2018, the FDA issued a safety communication announcing that codeine and hydrocodone cough and cold medications were no longer approved for use in children and adolescents aged 0 to 17 years.^3^ Using national data, we assessed whether these communications were associated with changes in prescriptions for codeine and hydrocodone cough and cold medications to children and adolescents.

## Methods

We conducted a cross-sectional analysis of 2014-2019 IQVIA Longitudinal Prescription Data, which includes all prescriptions dispensed from 92% of US retail pharmacies. Because the data were deidentified, the University of Michigan exempted this study from review. This report follows the Strengthening the Reporting of Observational Studies in Epidemiology (STROBE) reporting guidelines for observational studies.

Using an interrupted time series design, we evaluated the association between the 2 FDA safety communications and monthly prescriptions for codeine cough and cold medications to children and adolescents aged 0 to 17 years. We fitted a linear segmented regression model assessing for level and slope changes after April 2017 and January 2018.^5^ We repeated the analyses for children aged 0 to 11 years vs adolescents aged 12 to 17 years because the April 2017 FDA safety communication only affected the younger age group. To evaluate the association between the January 2018 FDA safety communication and monthly prescriptions for hydrocodone cough and cold medications to children and adolescents aged 0 to 17 years, we fitted a linear segmented regression model assessing for changes in level and slope after January 2018 only.

In all analyses, we excluded the months of the FDA safety communications. The conclusions were unchanged when also excluding the subsequent month. We included indicators for quarter to account for seasonal prescribing patterns. We used robust standard errors and also used Prais-Winsten estimators to account for first-order autocorrelation. Analyses used R version 4.0.3 (R Foundation for Statistical Computing) and 2-sided hypothesis tests with α = .05.

## Results

From 2014 through 2019, there were 1 145 357 prescriptions for codeine and hydrocodone cough and cold medications to children and adolescents aged 0 to 17 years. Of these, 577 524 (50.4%) were for females and 680 101 (59.4%) were for adolescents aged 12 to 17 years (Table). Prescriptions were most frequently written by family medicine physicians (331 472; 28.9%), pediatricians (254 659; 22.2%), and nurse practitioners (120 559; 10.5%).

**Table.** Characteristics of US Children and Adolescents Aged 0 to 17 Years With Dispensed Prescriptions for Codeine or Hydrocodone Cough and Cold Medications

Outcome	No. (%)^a^					
	2014	2015	2016	2017	2018	2019
No. of dispensed prescriptions^b^						
Total	364 641	283 771	207 433	152 151	82 121	55 240
Codeine	232 140 (63.7)	192 712 (67.9)	138 119 (66.6)	93 305 (61.3)	42 041 (51.2)	23 008 (41.7)
Hydrocodone	132 501 (36.3)	91 059 (32.1)	69 314 (33.4)	58 846 (38.7)	40 080 (48.8)	32 232 (58.3)
Age group, y						
0-11	161 173 (44.2)	119 251 (42.0)	84 103 (40.5)	53 202 (35.0)	27 707 (33.7)	19 820 (35.9)
12-17	203 468 (55.8)	164 520 (58.0)	123 330 (59.5)	98 949 (65.0)	54 414 (66.3)	35 420 (64.1)
Sex						
Male	178 946 (49.5)	138 551 (48.8)	101 438 (48.9)	74 569 (49.0)	40 678 (49.5)	27 756 (50.2)
Female	184 115 (50.5)	143 914 (50.7)	104 883 (50.6)	76 732 (50.4)	40 907 (49.8)	26 973 (48.8)
Unknown	1580 (0.1)	1306 (0.5)	1112 (0.5)	850 (0.6)	536 (0.7)	511 (0.9)
US Census region of patient residence						
Midwest	58 782 (16.1)	44 227 (15.6)	33 026 (15.9)	24 706 (16.2)	13 596 (16.6)	9273 (16.8)
Northeast	24 518 (6.7)	22 000 (7.8)	16 764 (8.1)	12 159 (8.0)	7079 (8.6)	4462 (8.1)
South	161 673 (44.3)	119 976 (42.3)	91 274 (44.0)	71 927 (47.3)	40 181 (48.9)	28 274 (51.2)
West	91 667 (25.1)	82 114 (28.9)	56 081 (27.0)	36 904 (24.3)	17 750 (21.6)	10 940 (19.8)
Puerto Rico or unknown	28 001 (7.7)	15 454 (5.4)	10 288 (5.0)	6455 (4.2)	3515 (4.3)	2291 (4.1)
Method of payment for prescription						
Cash	48 747 (13.3)	39 908 (14.1)	31 437 (15.2)	25 471 (16.7)	22 613 (27.5)	18 709 (33.9)
Medicaid	109 549 (30.0)	86 302 (30.4)	62 799 (30.3)	42 644 (28.0)	15 442 (18.8)	6007 (10.9)
Medicare^c^	1747 (0.5)	1563 (0.6)	1461 (0.7)	1227 (0.8)	622 (0.8)	486 (0.9)
Private insurance	202 278 (55.5)	153 339 (54.0)	110 087 (53.1)	81 294 (53.4)	41 614 (50.7)	27 949 (50.6)
Unknown	2320 (0.6)	2659 (0.9)	1649 (0.8)	1515 (1.0)	1830 (2.2)	2089 (3.8)

^a^ Based on 2014-2019 IQVIA Longitudinal Prescription Data, which includes all prescriptions dispensed from 92% of US retail pharmacies. Percentages may not equal 100.0% because of rounding errors.

^b^ The included prescriptions were for medications that combine codeine or hydrocodone with drugs such as guaifenesin, chlorpheniramine, and pseudoephedrine.

^c^ Covers children with end-stage kidney disease and certain children with disabilities.

Prescriptions for codeine cough and cold medications declined from 232 140 in 2014 to 23 008 in 2019 (−90.1%). The April 2017 and January 2018 FDA safety communications were not associated with changes in level or slope overall, among children aged 0 to 11 years, or among adolescents aged 12 to 17 years (Figure, A). The level change associated with the April 2017 communication was −2308 prescriptions (95% CI, −6417 to 1780 prescriptions) and the slope change was 389 prescriptions/month (95% CI, −714 to 1493 prescriptions/month). The level change associated with the January 2018 communication was −2018 prescriptions (95% CI, −6165 to 2129 prescriptions) and the slope change was −155 prescriptions/month (95% CI, −1268 to 958 prescriptions/month).

**Figure.**
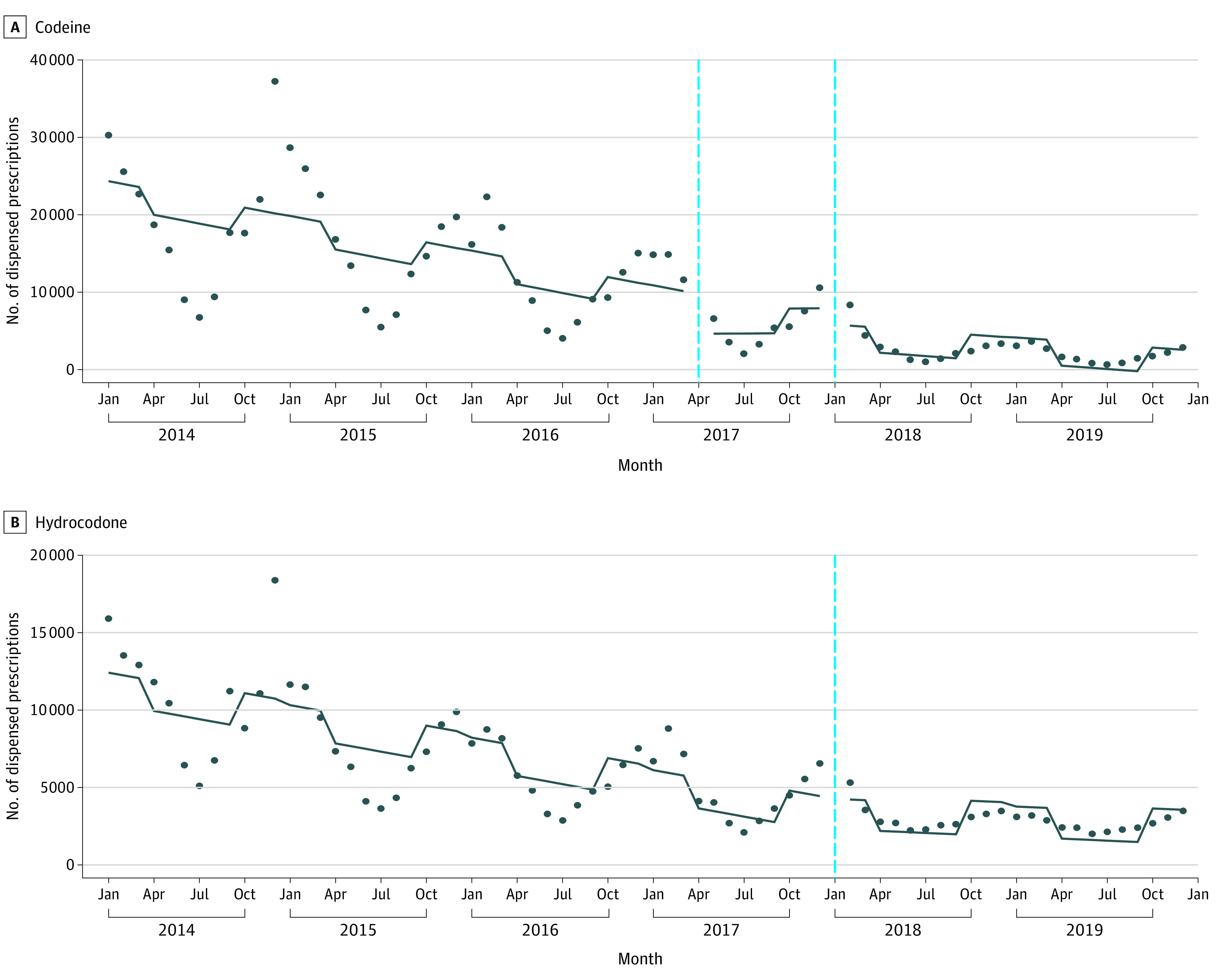
Monthly Number of Prescriptions for Codeine and Hydrocodone Cough and Cold Medications Dispensed to US Children and Adolescents Aged 0 to 17 Years Based on 2014-2019 IQVIA Longitudinal Prescription Data, which includes all prescriptions dispensed from 92% of US retail pharmacies. The included prescriptions were for medications that combine codeine or hydrocodone with drugs such as guaifenesin, chlorpheniramine, and pseudoephedrine. Graphs display fitted lines from linear segmented regression models. A, The left vertical line corresponds to the April 2017 safety communication from the US Food and Drug Administration announcing a contraindication against the use of codeine in children aged 0 to 11 years. The right vertical line corresponds to the January 2018 Food and Drug Administration safety communication announcing that codeine and hydrocodone cough and cold medications were no longer approved for use in children and adolescents aged 0 to 17 years. B, The vertical line corresponds to the January 2018 Food and Drug Administration safety communication.

Prescriptions for hydrocodone cough and cold medications declined from 132 501 in 2014 to 32 232 in 2019 (−75.7%). The January 2018 FDA safety communication was not associated with a level change but was associated with a slope increase (Figure, B). The level change associated with this communication was 109 prescriptions (95% CI, −1559 to 1777 prescriptions) and the slope change was 133 prescriptions/month (95% CI, 34 to 232 prescriptions/month).

## Discussion

Between 2014 and 2019, prescriptions to children and adolescents declined 90.1% for codeine cough and cold medications and 75.7% for hydrocodone cough and cold medications. The April 2017 and January 2018 FDA safety communications were not associated with changes in prescriptions for codeine cough and cold medications. For hydrocodone cough and cold medications, the January 2018 FDA safety communication was associated with only a slight deceleration in the rate of decline in prescriptions.

The findings contrast with studies showing that other safety communications, including a 2013 FDA communication contraindicating codeine use in children undergoing tonsillectomy, substantially decreased prescribing.^4,6^ A potential explanation is that clinicians were already aware of the risks of opioid cough and cold medications, perhaps because of the 2013 FDA communication and national campaigns to decrease pediatric codeine use.^2^ Even though the decline is impressive, approximately 55 000 prescriptions for opioid cough and cold medications were dispensed to children and adolescents in 2019, suggesting efforts to eliminate these prescriptions may still be needed.

A limitation is that the study period preceded the COVID-19 pandemic. Owing to decreased respiratory infections, prescriptions for opioid cough and cold medications to children and adolescents may have decreased further during the pandemic. In addition, the database did not capture all US prescriptions.
